# Targeting FABP4/UCP2 axis to overcome cetuximab resistance in obesity-driven CRC with drug-tolerant persister cells

**DOI:** 10.1016/j.tranon.2025.102274

**Published:** 2025-01-16

**Authors:** Yi-Chiao Cheng, Ming-Yao Chen, Vijesh Kumar Yadav, Narpati Wesa Pikatan, Iat-Hang Fong, Kuang-Tai Kuo, Chi-Tai Yeh, Jo-Ting Tsai

**Affiliations:** a Graduate Institute of Clinical Medicine, College of Medicine, Taipei Medical University, Taipei 110, Taiwan; b Division of Colon and Rectal Surgery, Department of Surgery, Tri-Service General Hospital, National Defense Medical Center, Taipei 114202, Taiwan; c Division of Gastroenterology and Hepatology, Department of Internal Medicine, School of Medicine, College of Medicine, Taipei Medical University, Taipei 110, Taiwan; d Division of Gastroenterology and Hepatology, Department of Internal Medicine, Shuang Ho Hospital, New Taipei City 23561, Taiwan; e Division of Urology, Department of Surgery, Faculty of Medicine, Universitas Gadjah Mada, Yogyakarta 55281, Indonesia; f Department of Medical Research & Education, Taipei Medical University–Shuang Ho Hospital, New Taipei City 23561, Taiwan; g Continuing Education Program of Food Biotechnology Applications, College of Science and Engineering, National Taitung University, Taitung 95092, Taiwan; h Department of Surgery, Division of Thoracic Surgery, Taipei Medical University Shuang-Ho Hospital, New Taipei City 23561, Taiwan; i Department of Radiology, School of Medicine, College of Medicine, Taipei Medical University, Taipei 110, Taiwan; j Department of Radiology, Taipei Medical University–Shuang Ho Hospital, New Taipei City 23561, Taiwan

**Keywords:** Colorectal cancer, Cetuximab resistance, FABP4/UCP2 Axis, Tumor microenvironment, Adipocytes, Drug-tolerant persister cells

## Abstract

Colorectal cancer (CRC) is closely linked to obesity, a condition that significantly impacts tumor progression and therapeutic resistance. Although cetuximab, an EGFR-targeting monoclonal antibody, is a cornerstone in metastatic CRC treatment, resistance often emerges, leading to poor outcomes. This study investigated the role of drug-tolerant persister (DTP) cells and their metabolic interactions within the tumor microenvironment (TME) in cetuximab resistance. Using patient-derived organoids and in vivo models, we identified the FABP4/UCP2 axis as a critical mediator of resistance. Organoids derived from cetuximab non-responders revealed upregulated FABP4 and UCP2 expression post-treatment. Coculture experiments with adipocytes showed that FABP4 and UCP2 promote lipid metabolic reprogramming, facilitating cancer cell survival in a dormant state. CRISPR/Cas9 mediated inhibition of FABP4 disrupted this metabolic interaction, sensitising resistant cells to cetuximab. In vivo, the FABP4 inhibitor BMS309403, either alone or in combination with cetuximab, significantly reduced tumor growth in resistant CRC models, highlighting its therapeutic potential. These findings establish the FABP4/UCP2 axis as a pivotal driver of cetuximab resistance in obesity-associated CRC and suggest that targeting this metabolic pathway could improve outcomes in DTP-resistant CRC patients.

## Highlights

• Leveraging the FABP4/UCP2 metabolic pathway as a therapeutic strategy.
• Overcome cetuximab resistance in obesity-associated colorectal cancer.
• Adipocyte co-culture activates the FABP4/UCP2 axis, inducing cetuximab tolerance in colorectal cells.

## Graphical abstract

**Figure:**
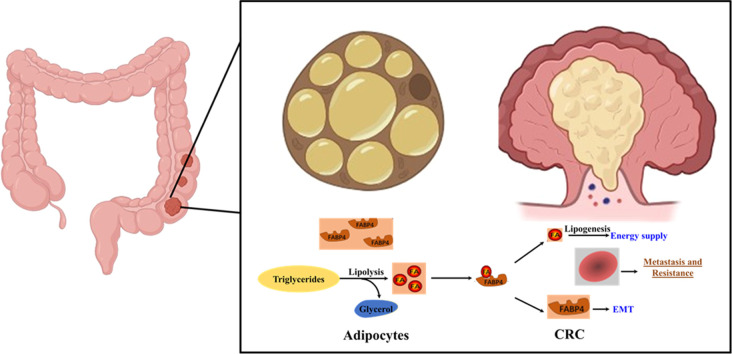

## Introduction

Colorectal cancer (CRC) is the third leading cause of cancer-related deaths globally [1]. Metastatic CRC (mCRC) accounts for approximately half of all new cases of CRC and has a dismal prognosis. Epidermal growth factor receptor (EGFR) plays a key role in cellular processes such as proliferation, differentiation, and survival, and therefore, EGFR-targeted therapies are crucial. Cetuximab, a common antibody (mAb) targeting EGFR has demonstrated survival benefits in patients with mCRC. Although anti-EGFR therapies and chemotherapy are effective against mCRC, the potential for cancer cells to develop drug resistance limits their practical applicability. The therapeutic effect of anti-EGFR mAbs typically diminishes after 8–10 months, with approximately 80 % of initially responsive patients developing resistance over time [2,3]. Non-genetic factors have increasingly been associated with treatment resistance, and such factors present a considerable challenge in cancer treatment. Drug-tolerant persister (DTP) cells are gaining recognition as crucial players in tumor non-genetic heterogeneity, and they have been observed across various cancers after the implementation of chemotherapy and targeted therapies. Therefore, DTPs have potential value for identifying a means of managing drug resistance before it becomes permanent due to genetic mutations. These cells, which are often in a quiescent or slow-cycling state, represent a small fraction of the original tumor. In addition, although DTPs may resume proliferation in vitro after drug removal, their offspring remain susceptible to the initial treatment, indicating such cells have transient rather than genetically inherited resistance [[4], [5], [6]].

A tumor may have intratumoral heterogeneity, that is, it may contain distinct subpopulations of cells with genetic or epigenetic variations. Such heterogeneity has critical implications for cancer treatment because different clones within a tumor may respond differently to therapy [6]. The tumor microenvironment (TME) influences both tumor behaviour and sensitivity to treatment. Cellular stresses can alter the TME and therefore potentially contribute to cancer persistence. Although research on the involvement of the TME in DTP cells is limited, various components of the TME have been identified as influencing tumor properties relevant to DTPs. Cancer-associated fibroblasts (CAFs) are a prominent component of the TME that are known to mediate drug resistance through the secretion of growth factors and chemokines [7]. Paracrine factors derived from CAFs influence cancer cell behaviours such as quiescence, stemness, and epithelial-mesenchymal transition (EMT) [8]. The interaction between cancer cells and CAFs through paracrine signaling pathways contributes to treatment resistance, particularly resistance to EGFR TKIs, by activating the IGF-1R pathway and downstream EMT and stemness signaling [9]. Inflammatory CAF-dominant stroma may induce an EMT phenotype in cancer cells by modulating Wnt signaling pathways, depending on the equilibrium between different CAF subtypes [10]. Research indicates that analyzing tumor-associated macrophages (TAMs) is crucial in elucidating the impact of TMEs on cancer cell behaviour. TAMs with an anti-inflammatory M2-like phenotype release growth factors, anti-inflammatory cytokines, and immune-suppressive molecules, contributing to tumor progression and treatment resistance [11]. TAMs and CAFs engage in reciprocal interactions, with CAFs attracting macrophages through the expression of stromal cell-derived factor 1 (SDF-1/CXCL12). SDF-1 promotes macrophage M2 polarization, which is characterized by increased IL-10 production [12].

Obesity has been established as a significant risk factor for the onset and progression of CRCs. In Europe, approximately 10 % of CRC cases are associated with overweight or obesity [13]. In a mouse xenograft model, a high-fat diet was demonstrated to induce tumor development; progression, including EMT; and inflammation in colon cancer [14]. Adipocytes are key components of the TME and are associated with processes such as metastasis, angiogenesis, and cell survival. Cancer-associated adipocytes (CAAs) represent a diverse group of cells within the TME, including intratumoral adipocytes, peritumoral adipocytes, recruited adipocytes, and de novo MSCs transformed into adipocytes or adipocyte-like cells that are capable of storing substantial amounts of high-energy fats [15]. When differentiated adipocytes interact with breast cancer cells, they undergo cellular changes, leading to the formation of CAFs, which are known to have immune-suppressive properties in humans. CAAs exert adverse effects on the TME because they release molecules such as adipokines, hormones, and inflammatory mediators (e.g., CCL6, CCL2, CCL5, MMP, VEGF, TNF, insulin, and leptin), which contribute to cancer proliferation and immune resistance. Several studies have indicated that CAAs have proinflammatory characteristics and therefore further exacerbate TME dysfunction [16]. In addition, research indicates that in cancer cells, lipolysis and lipogenesis may occur concurrently, and the presence of adipocytes in the cellular environment influences the reprogramming of lipid metabolism in cancer cells [17]. When cancer cells are cocultured with adipocytes, they exhibit increased expression of cluster of differentiation 36 (CD36), leading to increased absorption of fatty acids (FAs) by the cancer cells. Consequently, the FAs are oxidized in the mitochondria of the cancer cells and fuel cell proliferation, survival, invasion, metastasis, and resistance to treatment. These findings underscore the intricate interaction between adipose tissue and cancer cells, which can increase FA oxidation and cancer aggressiveness [17,18]. Free FAs produced by adipocytes play a crucial role in supporting tumor development, including structural reorganization and EMT [19]. FA-binding proteins (FABPs) constitute a family of small cytoplasmic proteins involved in lipid transport and binding within cells. These proteins facilitate the transfer of insoluble lipids across cellular components [20]. FABP4, which is abundant in adipose tissue and macrophages, plays a significant role in metabolic syndrome, inflammation, atherosclerosis, and other diseases in that it interacts with long-chain FAs and facilitates their delivery. Research indicates that the expression FABP4 is higher in cancer cells with metastatic potential. Notably, FABP4 expression has been reported to be elevated at the interface between ovarian cancer cells and adipocytes, and its mRNA and protein levels have been reported to be increased in prostate and breast cancer cells [21].

Nonmalignant cells, such as adipocytes, constitute a considerable portion of the tumor mass, and their presence is often associated with rapid growth and invasiveness. Understanding the reciprocal interactions between cancer cells and adipocytes is crucial to designing effective cancer treatments. Because cancer cells and tissues have higher lipid requirements than normal cells and tissues do, they develop mechanisms to enhance lipid absorption. For example, cancer cells increase their lipid uptake by upregulating plasma lipid receptors on their cell surfaces. CD36 is one such cell surface receptor that aids in the absorption of lipids from the extracellular environment [15]. Additionally, overexpression of FABPs can increase FA transport and enhance lipid absorption in cancer cells. Different isoforms of FABPs synthesized in various tissues play distinct roles in the regulation of FA uptake and cancer development. Research using breast cancer and glioblastoma cell lines indicated that hypoxia increases absorption of extracellular FAs by upregulating FABP3 and FABP7. Additionally, FABP5 overexpression promotes prostate cancer growth and proliferation, likely by enhancing the activity of intracellular receptors such as PPAR-γ. In vitro studies have demonstrated that genetic knockdown or silencing of FABP5 inhibits prostate cancer cell proliferation and invasiveness and that inhibitors targeting FABP5 exert synergistic effects with taxanes, leading to decreased tumor growth and metastasis [21]. mCRC represents the most common initial presentation among patients with CRC and is characterized by a poor prognosis. Cetuximab, a widely used monoclonal antibody (mAb) targeting EGFR, has demonstrated efficacy in improving survival rates for patients with mCRC. However, the practical utility of cetuximab is restricted by the emergence of drug resistance in cancer cells, which leads to short treatment responses and eventual resistance in the majority of responders to such treatment. DTP cells are an increasing subpopulation of tumor cells with nongenetic heterogeneity, and they can hinder the success of cancer treatment with chemotherapy and targeted medicines. These cells are typically dormant or slow cycling. However, they continue to proliferate after drug withdrawal, although their progeny remains sensitive to the initial treatment, indicating their resistance is transient. The findings of the current study elucidated the pivotal role of adipose tissue within the TME in influencing cancer progression and treatment resistance, particularly in the context of obesity—a significant risk factor for CRC. In our study, notable upregulation of FABP4 and UCP2 was observed in patients with CRC exhibiting poor responses to cetuximab, with a higher body mass index correlated with increased FABP4 levels. This association underscores the substantial influence of adipose tissue on CRC progression through fat accumulation. Our finding of increased presence of adipocytes within the TMEs of cetuximab-resistant patients, along with simultaneous upregulation of FABP4 and UCP2, highlights the potential role of these genes in conferring treatment resistance. Our combined in silico, in vitro, and in vivo studies further confirmed that adipocytes may promote cetuximab tolerance through the FABP4/UCP2 pathway by modulating lipid metabolism. This interaction not only promotes the proliferation of cetuximab-resistant DTP cells but also underscores the complexity of the TME interactions contributing to the adaptive resistance mechanisms in CRC.

In summary, this study highlighted the pivotal role of the oncogenic FABP4/UCP2 axis and its association with unfavourable treatment outcomes, particularly in the context of obesity-related CRC. Through the identification and characterization of interactions between cancer cells and TME components, particularly adipocytes, our study reveals a potential avenue to overcoming resistance in cetuximab-treated CRC, potentially transforming the treatment landscape for this challenging disease.

## Materials and methods

### Tissue processing for organoid derivation

Tissue samples were procured from pathological sources and reconstituted in DMEM/F12 media enriched with 1x GlutaMAX, penicillin-streptomycin, and HEPES, collectively called “complete media”. Tissue samples were obtained from adult patients undergoing surgery, following the ethical guidelines set forth by the Tri-Service General Hospital Institutional Review Board (TSGHIRB No.: A202405118). These clinical specimens were then immersed in these medium, and healthy tissue segments were finely diced after removing any adherent fat or muscle tissue. Fragments measuring approximately 5 mm³ were maintained at 20 °C for subsequent DNA isolation. The remaining tissue samples underwent organoid derivation; 2–4 pieces were fixed in formalin for histological and immunohistochemical analysis. The samples were then incubated in a solution containing 0.125 % trypsin in complete media at 37 °C to facilitate tissue digestion. During the incubation period, the tissue suspension was intermittently agitated every 10 min by using a 1-mL pipette, with the total incubation time not exceeding 60 min. Following digestion, the tissue suspension was filtered using a tissue strainer, diluted with complete media, and centrifuged at 300×g. The pellet was then resuspended in ice-cold water. Approximately 10,000 cells were resuspended in 40 μL of basement membrane extract (BME), and these cells were plated as 10-μL droplets on the bottoms of preheated suspension culture plates. After the BME was allowed to solidify for 30 min at 37 °C, a prewarmed organoid medium was added. During the initial week, a Rho-associated kinase (ROCK) inhibitor was incorporated into the medium to promote organoid proliferation.

### Organoid culture

The organoid medium was enriched with a combination of supplements, including B27, N-acetyl-l-cysteine, nicotinamide, human EGF, A83–01, human FGF10, human FGF2, prostaglandin E2, CHIR 99,021, forskolin, and 4 % (v/v) RSPO and Noggin, synthesized using the r-PEX protein expression platform. On days 7 and 14, the organoids were divided into two cohorts. They were detached from the plate, rinsed with complete media and dissociated in recombinant trypsin at 37 °C. Frequent pipetting every 5 min was used to facilitate the disruption process. Once a single-cell suspension was obtained, trypsin action was halted through the addition of complete media. The cells were then resuspended in 70 % BME and organoid media, replated, and immediately treated with Y-27,632 to boost the growth of new organoids. The organoids were divided every 1–2 weeks, with media changes performed every 2–3 days.

### Immunohistochemical staining

Clinical samples of CRC had been previously fixed in 10 % formalin and embedded in paraffin. Following this, the embedded specimens were deparaffinized and rehydrated. The deparaffinized slides were subjected to antigen retrieval, and endogenous peroxidase activity was blocked by incubating them with 1 % hydrogen peroxide in PBS for 30 min. Subsequently, the slides were exposed to anti-FABP4 (1:1000), anti-UCP2 (1:1000), anti-UCP2 (1:1000), anti-FASN (1:1000), anti-Ki-67 (1:1000), anti-KRAS (1:1000), and anti- PPAR-γ (1:1000). Following incubation, the slides were washed and incubated with biotinylated link universal antiserum for 1 h at room temperature. The slides were then washed and stained using the chromogen 3, 3-diaminobenzidine hydrochloride. Finally, the slices were washed with double distilled water, counterstained with Mayer's hematoxylin, and mounted with Permount mounting media for microscopic observations.

### Single-cell sample preparation and scRNA-sequencing

After the CRC specimens were thawed, they were immediately washed to eliminate as much DMSO as possible. A minimum viability of 90 % was ensured before cell isolation commenced. Dead cells and debris were eliminated from all samples by using a dead cell removal kit. Subsequently, single-cell suspensions, complementary DNA, and single-cell libraries were prepared using the Chromium single-cell v2 3 kits (10× Genomics) following the manufacturer's instructions. RNA sequencing was conducted on a NextSeq 550 at the core facility of the National Defense Medical Center by using a single RNA sequencer. For data analysis, including GEO data collection and differential gene expression analysis (DEGs), we employed the public dataset GSE82236, which comprises RNA-seq data from both cetuximab-sensitive and cetuximab-resistant colon cancer samples. A comprehensive analysis was performed using the R DSeq2 package.

### Cell lines and reagents

The CRC cell lines DLD-1 and HT-29 were cultured in DMEM medium supplemented with 10 % fetal bovine serum (FBS) and 2 mM l-glutamine (Life Technologies) at 37 °C in a 5 % CO_2_/95 % air humidified atmosphere. Similarly, patient-derived CRC cells were cultures in RPMI-1640 medium supplemented with 10 % FBS under identical conditions. The mouse C26 Tumor Cell Line was obtained from Cellonco (CAT#: IOC-ZP090). The FABP4 inhibitor, procured from MecChemExpress Taiwan (BMS309403, Cat. No.: HY-101,903), was dissolved in DMSO to create a stock solution of 10 mM, which was stored at −20℃. Erbitux, the active ingredient of cetuximab, was provided by Shuang Ho Hospital (Taiwan) and was originally purchased from Merck KGaA (Darmstadt, Germany). Unless otherwise specified, FOLFOX-surviving colon cancer cells were generated by incubating DLD1 or HT-29 cells with a combination of 50 µM 5-FU and 1.25 µM oxaliplatin (FOLFOX) for 48 h. After treatment, the adherent cells that survived the FOLFOX exposure were subjected to trypsin/EDTA treatment. In some experiments, FOLFOX-resistant colon cancer cells were utilized. These cells were developed by exposing DLD1 or HT-29 cells to FOLFOX at clinically relevant doses and schedules over 12 cycles, each lasting one week. Initially, the cells were treated with FOLFOX (25 µM 5-FU and 0.625 µM oxaliplatin) for 72 h. The surviving cells were then cultured in drug-free medium for 4 to 5 days. This cycle was repeated 12 times. Subsequently, the surviving cells were exposed to higher doses of FOLFOX (50 µM 5-FU + 1.25 µM oxaliplatin or 100 µM 5-FU + 2.5 µM oxaliplatin) for 2 to 3 days per week over approximately 4 weeks. Finally, the resistant cells were maintained in a normal culture medium containing a low dose of FOLFOX (5 µM 5-FU + 0.125 µM oxaliplatin).

### Western blotting

In total, 40 mg of proteins were extracted from total cell lysate and separated through SDS-PAGE before they were transferred to a polyvinylidene difluoride membrane. Subsequently, the blot was blocked using a 5 % skim milk solution, and the proteins of interest were identified through incubation with primary antibodies. List of antibodies and dilution used in Supplementary Table S1. The membrane was washed and incubated with HRP-conjugated secondary antibodies. Finally, protein bands were visualized using Kodak X-Omat Blue film and enhanced chemiluminescence reagents from Perkin Elmer.

### Immunofluorescence microscopy

The cells were cultured on coverslips, followed by fixation with 4 % paraformaldehyde, permeabilization, and labeling with Alexa Fluor 488 Phalloidin (Life Technologies). Subsequently, images of the slides were captured and observed using a Zeiss Axiophot fluorescence microscope. Additionally, the nuclei were counterstained with 4′,6-diamino-2-phenylindole.

### Molecular cloning

All constructs were generated using standard protocols. Genes, including FABP4 and UCP2, and their fragments, were isolated through polymerase chain reaction (PCR) amplification by using specific primers. The resulting PCR products were cloned into the pCDNA3.1 vector (Invitrogen).

### RNA isolation, reverse-transcription PCR analysis

RNA was extracted using TRIzol (Invitrogen), and its quality was assessed by measuring the A260/A280 ratio and through agarose gel electrophoresis and UV–visualization after ethidium bromide staining. For reverse transcription, 5 µg of RNA was mixed with MMLV buffer, dNTPs, primer, and MMLV reverse transcriptase (Promega). The reaction was initiated at 37 °C and terminated at 70 °C. A portion of the resulting cDNA product was then amplified through PCR.

### CRISPR-Cas9 knockout

Cells lacking FABP4 were created using CRISPR/Cas9 with a lentiviral vector. Specifically, sgRNA sequences were designed using the Benchling CRISPR tool and cloned into lentiCRISPRv2. 293T cells were co-transfected with this vector, psPAX2, and VSV-G by using PEI to produce lentivirus. After 48 h, the virus-rich supernatant was harvested and used to infect tumor cells in the presence of polybrene. Following infection, the cells were selected with puromycin. Finally, single clones exhibiting the most effective gene ablation was isolated and expanded.

### Intracellular and lipid reactive oxygen species detection assay

Intracellular and lipid reactive oxygen species were assessed in cancer cell lines by using commercial assay kits (Abcam) containing 2′,7′-dichlorofluorescein diacetate (DCFDA) and C11 BODIPY. Following 24-h drug treatment, with 75–100 µM tert‑butyl hydroperoxide serving as a positive control, approximately 100,000 cells per treatment were harvested and washed. These cells were then stained under the kit protocols, and the percentage of stained cells was determined using a C6 Accuri flow cytometer (Becton Dickinson, USA). Data analysis was performed using FlowJo v10.5.3.

### Preclinical in vivo validation

We obtained 24 8-week-old, female, nonobese diabetic (NOD)/severe combined immunodeficient (SCID) mice from BioLASCO Taiwan (Taipei, Taiwan) and housed them under standard experimental pathogen-free conditions. Additional NOD/SCID mice were obtained from the National Laboratory Animal Center in Taiwan and maintained under institutional policies. All animal procedures were approved by the Institutional Animal Care and Use Committee at Taipei Medical University. This xenograft study involving tumor-bearing mice was approved by the Institutional Laboratory Animal Committee of Taipei Medical University (approval number: LAC2023–0511). In vivo efficacy tumor assay was conducted; patient-derived organoids DTP (5 × 10^5^) suspended in 50 μL of PBS were subcutaneously inoculated into the abdominal fat pad of each mouse as a heterotopic implantation. Before inoculation, the mice were anaesthetized with xylazine (i.p, 8 mg/kg) and ketamine (20 mg/kg). The mice were randomly divided into the following four groups (six per group) that were subjected to different treatments: vehicle group (PBS P.O, 5 times/week), cetuximab alone (10  mg/kg, 5 times/week), FABP4 inhibitor alone (BMS309403 20 mg/kg, 5 times/week), and a combination of both drugs (both regimens). Throughout the experiment, the subcutaneous tumors were measured weekly by using calipers, and their volumes were calculated using a standard formula (1/2 × width × length^2^). At the end of the experiments, the mice were euthanized, and serum and tissues were harvested for further serological and histological examination, respectively. Furthermore, the lungs were extracted from the mice for histological analysis.

### Statistical evaluation

For comprehensive statistical analysis, we utilized two different software platforms: GraphPad Prism 6.0 (San Diego, CA) and IBM SPSS version 25.0 (Armonk, NY). To examine associations between categorical variables, Pearson's chi-square test was applied. For continuous variables, we performed paired Student's *t*-tests, one-way ANOVA, and multivariate analyses to assess differences within groups. Survival rate differences between groups were evaluated using Kaplan-Meier survival curves, which also provided probabilities for various events. A p-value of <0.05 was considered to indicate statistical significance.

## Results

### Establishment of an organoid model reflecting tissue architecture in cetuximab non-responders

**Fig. 1:**
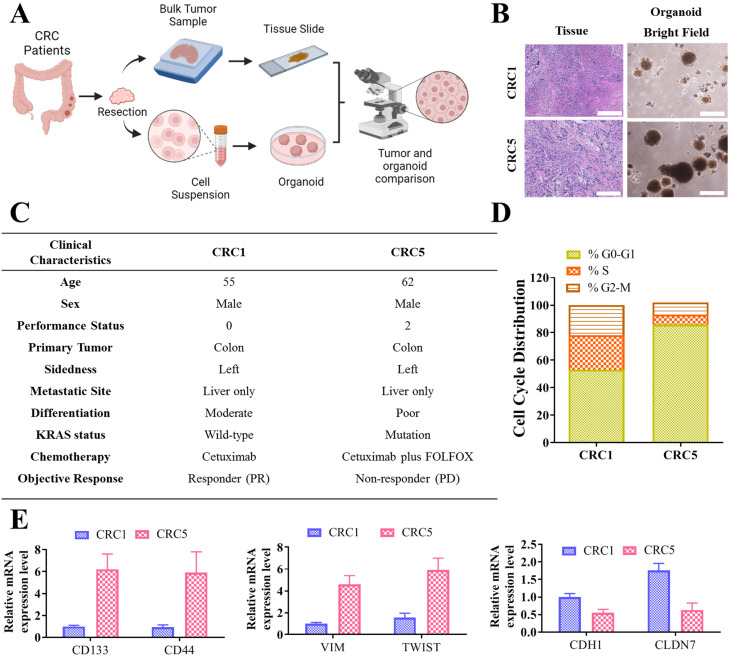
**Establishment of an organoid model reflecting the tissue architecture in cetuximab non-responder patients with CRC.** (A) Schematic of the steps of the methodology, from the initial collection of CRC bulk clinical specimens to the cultivation of organoids. (B) Cohort recruitment and organoid formation process. A total of five patients who exhibited responsiveness and non-responsiveness to cetuximab treatment were recruited from the National Defense Medical Center cohort, and two organoid cultures were successfully established. These organoids closely mimicked the architectural tissue organization of the original tumors and exhibited high expression of EGFR, a key marker of interest in this study (scale bar = 50 μm) (C) The clinical profiles of responder and non-responder patients with CRC highlighted features such as left-sided CRC, liver oligometastases, and KRAS wild-type or mutant status. Responders were defined as patients achieving a partial response (PR), while non-responders included those with progressive disease (PD). These patients had undergone prior treatment with doublet chemotherapy and anti-EGFR therapy, resulting in a stable response based on RECIST criteria. (D) Cell cycle analysis on samples from two patients from our cohort labelled CRC1 and CRC5. (E) qRT-PCR analysis was conducted to analyze the mRNA expression of stemness (CD133 and CD44) and mesenchymal (VIM and TWIST) and epithelial (CDH1 and CLDN7) markers.

To investigate the biological mechanisms underlying cetuximab resistance in CRC, we established a patient-derived organoid model that recapitulates the intricate tissue architecture observed in cetuximab non-responders. This model was developed through the processing of CRC clinical specimens, as illustrated in Fig. 1. The schematic presented in Fig. 1A depicts each step of the methodology that was used, from the initial collection of CRC bulk clinical specimens to the cultivation of organoids. This process enabled a direct comparison at the tissue level, underscoring the model's reliability in preserving tumor architecture. Fig. 1B presents the cohort recruitment and organoid formation process. Within the cohort from the National Defense Medical Center, we recruited a small subset of five patients who exhibited non-responsiveness to cetuximab treatment. From this group, we successfully established two organoid cultures. These organoids closely mimic the architectural tissue organization of the original tumors and exhibit a high expression of EGFR, a key marker of interest in this study (scale bar = 50 μm). The clinical profiles of the responder and non-responder patients (Patients that achieved partial response (PR) were categorized as responders, whereas those that exhibited progression on disease (PD) were considered non-responders were depicted in Fig. 1C, reveal characteristics such as left-sided CRC, liver oligometastatic, and KRAS wild-type and mutation status. Notably, these patients had previously received doublet chemotherapy and anti-EGFR co-treatment, with this treatment resulting in a stable disease response, according to RECIST criteria. To further elucidate the resistance mechanisms, we performed a cell cycle analysis on samples from two patients from our cohort, labelled CRC1 and CRC5, as shown in Fig. 1D. Fig. 1E illustrates the markers associated with DTPs, indicating increased expression of stemness (CD133 and CD44) and mesenchymal (VIM and TWIST) markers, along with reduced expression of epithelial (CDH1 and CLDN7) markers in the qRT-PCR analysis. These findings provide valuable insights into the cellular behaviour's underlying resistance mechanisms in our organoid models. The notable findings we obtained through our establishment of a high-fidelity patient-derived organoid model from cetuximab non-responder patients with CRC indicate that this model can provide a valuable tool that can assist with in-depth studies on cetuximab resistance to identify potential avenues for developing targeted therapeutic strategies for CRC.

### Co-expression of FABP4 and UCP2 following cetuximab treatment in high diapause-state persisters and residual CRC cells

**Fig. 2:**
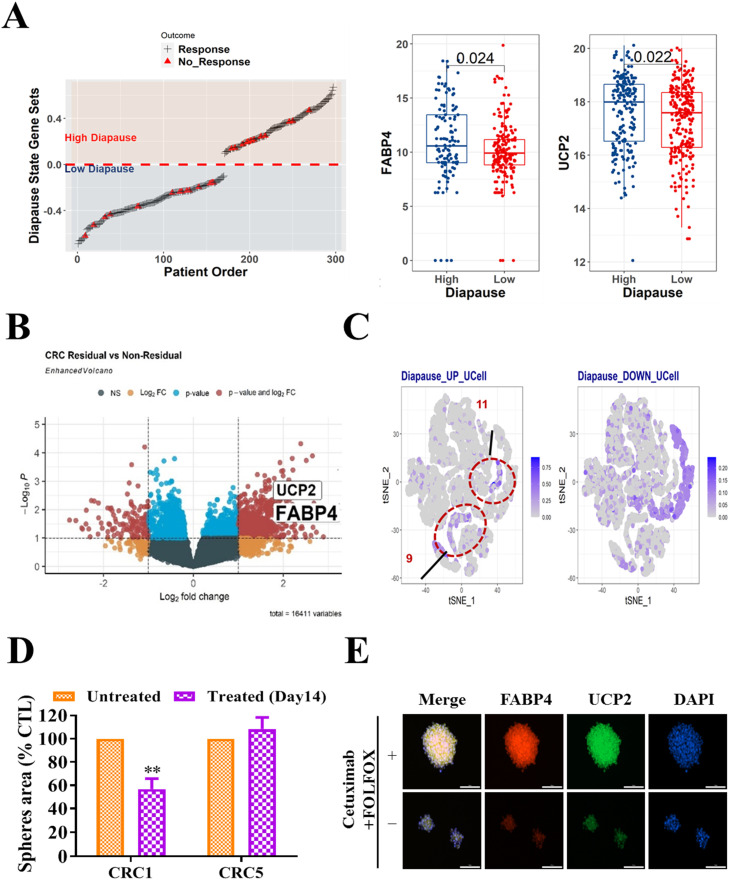
**FABP4 and UCP2 were co-expressed following cetuximab treatment in high diapause-state persisters and residual CRC cells. (A)** Analysis of the TCGA-COAD cohort based on the diapause-state signature score revealed elevated levels of FABP4 and UCP2 in patients with a high diapause-state-like phenotype during drug-tolerant persistence. **(B)** Patients with residual CRC exhibited significant upregulation of FABP4 and UCP2, indicating an association between their overexpression and poorer outcomes in CRC. **(C)** Single-cell profiling revealed high diapause signature scores in clusters with elevated levels of both FABP4 and UCP2, with particularly high scores noted for clusters 9 and 11. **(D)** Patient-derived CRC organoids treated with cetuximab for 14 days exhibited no significant change in viability, reflecting the clinical outcomes of non-responsive patients with CRC from whom the organoids were derived. **(E)** The PDTO1 organoid model as shown in the immunofluorescences image, exhibited increased co-expression of FABP4 and UCP2 after cetuximab treatment compared with that pretreatment as well as enhanced cellular viability, suggesting that targeting these markers could disrupt cetuximab tolerance in CRC. **: *p* < 0.01.

In Fig. 2, we illustrate the relationships between high diapause states, residual CRC cells, and organoids derived from patient CRCs, revealing co-expression of FABP4 and UCP2 following treatment with cetuximab. In the analysis of the TCGA-COAD cohort that was stratified by the diapause-state signature score, elevated levels of FABP4 and UCP2 were observed in patients with a high diapause-state-like phenotype during drug-tolerant persistence (Fig. 2A). Similarly, patients with residual CRC exhibited significant upregulation of both FABP4 and UCP2, indicating an association between their upregulation and poorer outcomes in CRC (Fig. 2B). At the single-cell level, clusters with elevated levels of both FABP4 and UCP2 had high diapause signature scores, with the scores being particularly notable in clusters 9 and 11 (Fig. 2C). Although the CRC organoids derived from patients treated with cetuximab for 14 days exhibited no significant change in viability, mirroring the clinical outcomes in non-responsive cases (Fig. 2D). Interestingly, one organoid model, PDTO1, the fluoresces image, exhibited increased co-expression of FABP4 and UCP2 after cetuximab treatment, suggesting that targeting these markers could disrupt cetuximab tolerance in CRC and thus enhance cellular viability (Fig. 2E; scale bar = 50 μm). This finding suggests that targeting FABP4 and UCP2 markers may be a promising avenue for enhancing treatment outcomes in CRC.

### Co-expression of FABP4, EGFR, and UCP2 in patients with CRC with poor responses to cetuximab

**Fig. 3:**
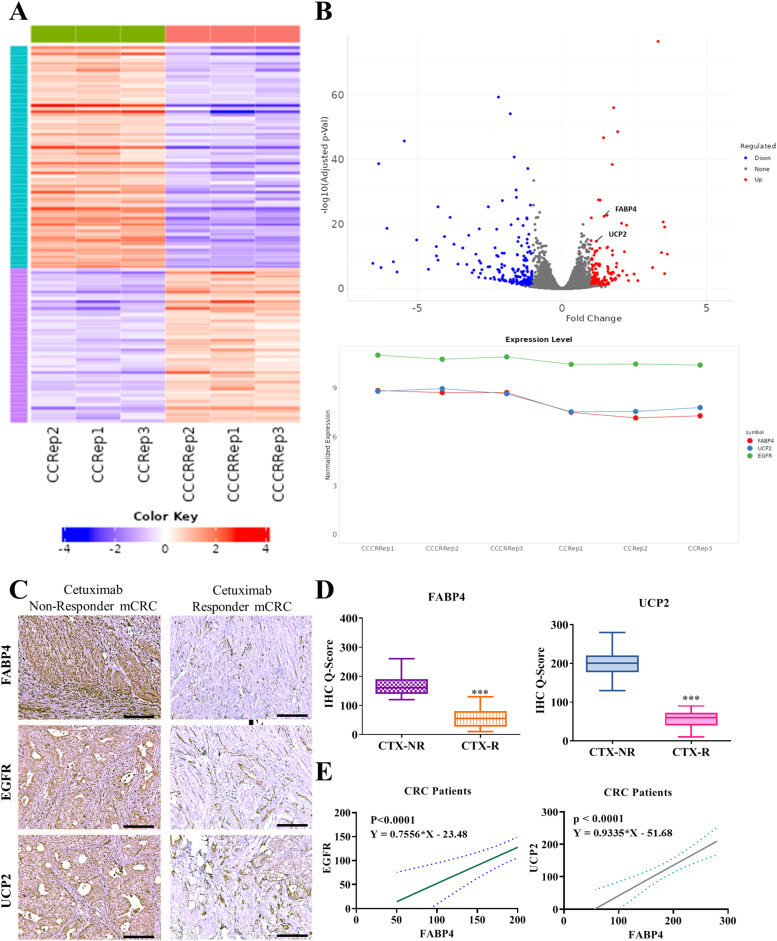
**High co-expression of FABP4 and UCP2 in clinical tumor tissue specimens from cetuximab non-responder patients with CRC. (A)** Differential gene expression analysis was conducted using the GEO dataset GSE82236, which comprises RNA-seq data from both cetuximab-sensitive (CC) and cetuximab-resistant colon cancer (CC—CR) samples, by using the R DSeq2 package. FABP4, UCP2, and EGFR were identified as the key differentially expressed genes (DEGs), as evidenced by their elevated expression levels in the cetuximab-resistant CRC samples compared with in the cetuximab-sensitive ones. The results for these DEGs are presented in a heatmap, volcano plot, and line graph, respectively (**A, B**). **(C)** IHC staining revealed elevated expression levels of FABP4, EGFR, and UCP2 in CRC tissue samples from patients with poor responses to cetuximab treatment**. (D)** Expression analysis revealed elevated levels of FABP4 and UCP2 in poor cetuximab responders compared with in responders (as represented by IHC-Q score; *n* = 5**). (E)** A significant correlation was noted between FABP4 and UCP2 expression in CRC tissue specimens, implicating the role of a significant axis in regulating clinical responses to cetuximab among patients with CRC. ***: *p* < 0.001.

In our previous result, we elucidated the molecular mechanisms underlying cetuximab resistance in patients with CRC, revealing a significant association between the co-expression of FABP4 and UCP2 and treatment outcomes. To further confirm the importance of FABP4 and UCP2, differential gene expression analysis (Fig. 3A) was conducted using the GEO dataset GSE82236, which comprises RNA-seq data from both cetuximab-sensitive (CC; *n* = 3) and cetuximab-resistant colon cancer (CC—CR; *n* = 3) samples. Through a comprehensive analysis using the R DSeq2 package, we identified FABP4, UCP2, and EGFR as key differentially expressed genes (DEGs), as evidenced by their elevated expression levels in cetuximab-resistant CRC samples compared with cetuximab-sensitive ones; the results for these DEGs are presented in a heatmap, volcano plot, and line graph, respectively (Fig. 3A). To identify key genes, KEGG pathway analysis was performed on differentially expressed genes (DEGs) between cetuximab non-responders and responders, revealing pathways associated with the regulation of lipolysis in adipocytes (Supplementary Figure S1). To further validate the selected DEGs, including FABP4 and UCP2, protein-protein interaction analysis was conducted using the STRING database (https://string-db.org/), demonstrating a strong association between FABP4 and UCP2 (Supplementary Figure S2). Additionally, qRT-PCR analysis confirmed their expression levels, as illustrated in the bar plot in Supplementary Figure S3. Immunohistochemical (IHC) analysis (Fig. 3B) was used for representative staining of CRC tissue samples from patients with poor responses to cetuximab treatment, with the results revealing elevated expression levels of FABP4, EGFR, and UCP2. This underscores their potential involvement in mediating resistance mechanisms. Expression analysis was conducted on clinical samples (Fig. 3C), which revealed significantly elevated levels of FABP4 and UCP2 in poor cetuximab responders compared with in responders (*n* = 10), indicating they are associated with reduced treatment efficacy. Further bar plot represents the IHC-Q score, representing induced expression of FABP4 and UCP2 in non-responder compared to responder patients. Therefore, FABP4 and EGFR expressions among patients with CRC might suggest a potential interaction or coregulatory mechanism that influences cetuximab response (Fig. 3D). Furthermore, a significant correlation was observed (*p* < 0.001) between FABP4 and UCP2 expression in CRC tissue specimens (Fig. 3E), highlighting the crucial role of the FABP4/UCP2 axis in regulating clinical responses to cetuximab treatment. The co-expression of FABP4 and UCP2, coupled with their association with EGFR expression, indicates the existence of a critical molecular axis that influences cetuximab resistance in patients with CRC. These findings highlight FABP4 and UCP2 as potential biomarkers for predicting cetuximab response and provide a basis for developing targeted therapeutic strategies to overcome resistance in CRC treatment.

### Abundance of large peritumoral adipocytes in the TME among cetuximab non-responders associated with FABP4 and UCP2 expression

**Fig. 4:**
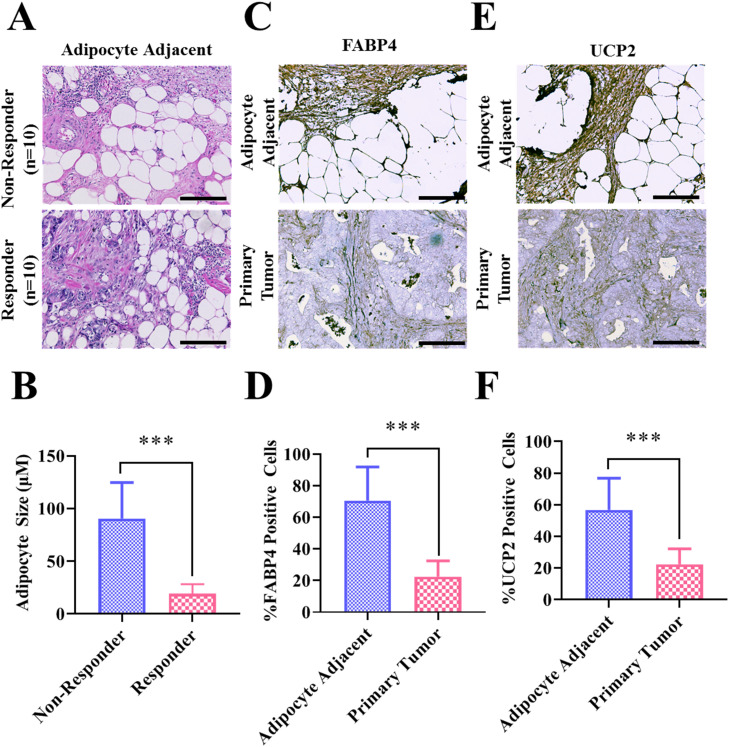
**The abundance of adipocytes with elevated FABP4 and UCP2 expression in the TME of cetuximab non-responder patients with CRC. (A)** H&E staining of the adjacent tumor area revealed an abundance of adipocytes in cetuximab non-responders. **(B)** The adipocyte size was significantly larger in cetuximab non-responders than in responders. (**C and E**) IHC staining demonstrated higher expression levels of FABP4 and UCP2 in areas adjacent to adipocytes compared with the primary tumor. (**D and F**) IHC scoring revealed a significantly higher proportion of positively stained cells expressing both FABP4 and UCP2 in the vicinity of adipocytes compared with the primary tumor. ***: *p* < 0.001.

In our investigation of patients with CRC who were nonresponsive to cetuximab treatment, a significant infiltration of adipocytes (fat cells) was observed in the TME. This indicates a potential interaction between cancer cells and adipocytes, potentially involving the proteins FABP4 and UCP2, which may contribute to treatment resistance (Fig. 4). In Fig. 4, we illustrate the adipocyte infiltration in the TME of patients with CRC who were nonresponsive to cetuximab. Hematoxylin and eosin (H&E) staining of the adjacent tumor area revealed an abundance of adipocytes in these non-responders (Fig. 4A). Furthermore, Fig. 4B indicates that the adipocytes in non-responders were significantly larger than those in patients who were responsive to cetuximab. Figs. 4C and 4E present the results of the IHC staining, which indicated that the areas adjacent to adipocytes exhibited higher expression levels of FABP4 and UCP2 than the primary tumor areas did. This observation was further substantiated in Fig. 4D and 4F, which present IHC scoring results suggesting significantly higher expression levels of both FABP4 and UCP2 in the cells adjacent to adipocytes than in the cells in the primary tumor. Notably, the TME in CRC non-responders was characterized by substantial infiltration of adipocytes, indicating elevated co-expression of FABP4 and UCP2. This observation suggests that the FABP4/UCP2 axis plays a pivotal role in the remodeling of treatment tolerance in patients with CRC, particularly against anti-EGFR treatments. It further underscores the potential for targeting the FABP4/UCP2 axis as a strategy for modulating the TME and countering treatment resistance in CRC.

### Up-regulation of the FABP4/UCP2 axis following in vitro coculture of cetuximab-tolerant CRC cells with adipocytes

**Fig. 5:**
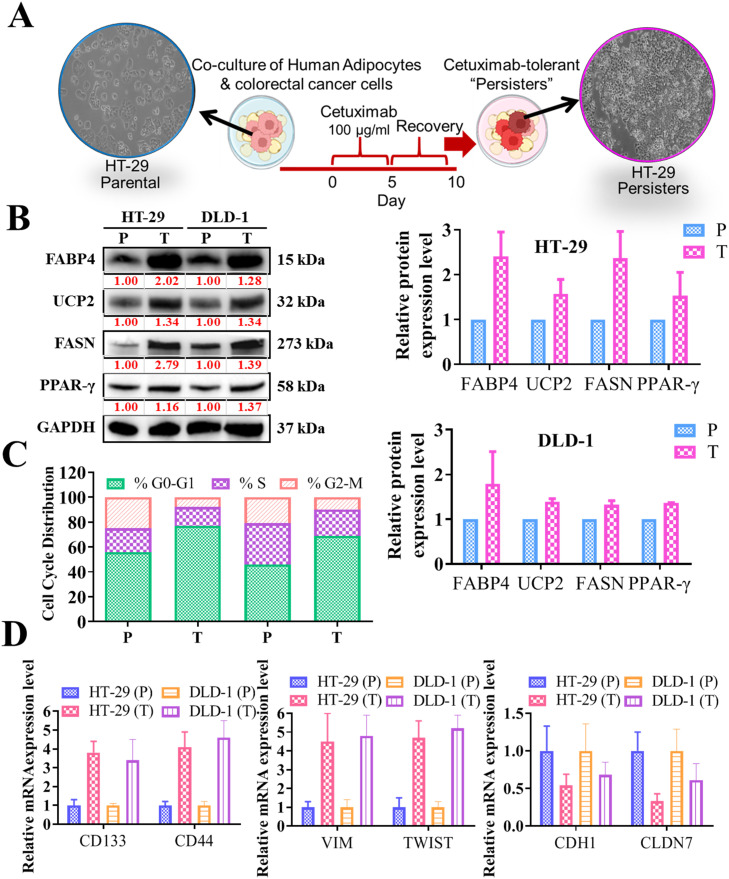
**Upregulation of FABP4/UCP2 axis following in vitro coculture of cetuximab-tolerant CRC cells with adipocytes. (A)** Schematic of the in vitro transformation of cetuximab-tolerant CRCs cells together with FOLFOX treatment (scale bar = 25 μm). **(B)** Western blot analysis demonstrates significantly high expression of FABP4 and its downstream effector UCP2 in cetuximab-tolerant clones compared with parental cells accompanied by increased expression of adipogenic markers (FASN and PPAR-γ). **(C)** PI-staining flow cytometry revealed a higher percentage of cells in the resting G_0_/G_1_ phase, indicating cell cycle arrest is associated with a diapause state in CRC persister cells. **(D)** qRT-PCR analysis demonstrated increased expression of stemness (CD133 and CD44) and mesenchymal (VIM and TWIST) markers and reduced expression of epithelial (CDH1 and CLDN7) markers. P: parental cells, T: cetuximab-tolerant persister cells.

After cetuximab and FOLFOX treatment, the DTP cells exhibited upregulation of FABP4 and UCP2 and entered a state of dormancy characterized by significantly decelerated cell cycle activity. This dormancy state functioned as a survival mechanism, enabling the cells to withstand the treatment. Fig. 5 presents a potential therapeutic approach involving the coculture of CRC cells and adipocytes in vitro. The protocol schema in Fig. 5A outlines the experimental setup. Notably, the coculture system resulted in notable differentiation of adipocytes, as depicted by the increased intensity of red oil O staining (Fig. 5A, macroscopic scale). This differentiation was associated with the acquisition of cetuximab tolerance in persister cells, underscoring the crucial role of adipocyte differentiation in generating cetuximab-tolerant clones (scale bar = 50 μm). Further, the upregulation of the FABP4/UCP2 axis (as depicted in the Western blot and quantification bar plot, Fig. 5B) and the subsequent upregulation of adipogenic markers (FASN and PPAR-γ; Fig. 5B) observed in the persister CRC cells during coculture with adipocytes underscores the importance of this axis in promoting adipogenesis within persister cells upon interaction with adipocytes. This process is integral to transforming persister cells into treatment-tolerant entities in the context of CRCs, as evidenced by PI-staining flow cytometry indicating a higher percentage of cells in the resting G0/G1 phase, suggesting a slowed cell cycle response of cetuximab persister cells in CRC (Fig. 5C). The current study re-examined the markers associated with DTPs through qRT-PCR analysis (Fig. 5D), and the results revealed increased expression of stemness (CD133 and CD44) and mesenchymal (VIM and TWIST) markers and reduced expression of epithelial (CDH1 and CLDN7) markers. This study demonstrated that during coculture with adipocytes, FABP4 in CRC cells induced the generation of persister cells following cetuximab treatment (Fig. 5). This finding indicates that targeting FABP4 is a promising strategy for inhibiting the acquisition of treatment tolerance.

### Co-culture of colorectal cells with adipocytes leading to activation of the FABP4/UCP2 axis and tolerance to cetuximab treatment

**Fig. 6:**
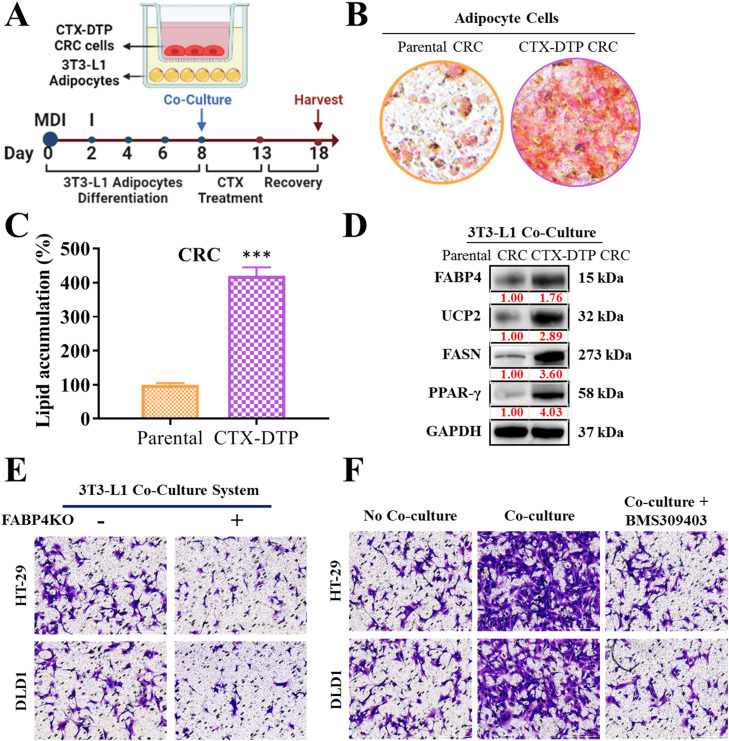
**Coculture of CRC cells with adipocytes leading to activation of the FABP4/UCP2 axis and tolerance to cetuximab treatment. (A)** Illustration of the experimental setup for coculturing CRC cells and adipocytes. Enhanced adipocyte differentiation, as evidenced by the increased intensity of red oil O staining indicated in both macroscopic views **(B)** and quantitative analysis **(C)**, is associated with cetuximab-resistant persister cells. **(D)** Upregulation of FABP4/UCP2 and adipogenic markers (FASN, PPAR-γ) in cocultured persister CRC cells underscores the importance of this axis in promoting resistance. (E) Knockdown of FABP4 in cocultured CRC cells significantly reduces the invasive capacity of cetuximab-resistant persister cells, underscoring FABP4 as a promising therapeutic target. (F) Combined treatment with the FABP4 inhibitor BMS309403 and cetuximab diminishes the invasive capacity of cetuximab-resistant persister cells, suggesting the potential to overcome adipocyte-induced resistance. ***P < 0.001.

The schematic protocol for the in vitro co-culture experiment of CTX-DTP CRC cells and 3T3-L1 adipocytes is illustrated in Fig. 6A. The CTX-DTP CRC cells cocultured with adipocytes exhibited significant activation of the FABP4/UCP2 signaling axis, which was closely associated with the development of resistance to cetuximab treatment. This co-culture environment appeared to enhance the metabolic and molecular interactions mediated by the FABP4/UCP2 axis pathway, contributing to the ability of CTX-DTP CRC cells to withstand the cytotoxic effects of cetuximab in Fig. 6B and C. Notably, adipocyte differentiation, as evidenced by the higher intensity of red oil O staining (macroscopic scale in Fig. 6B, with its quantification shown in Fig. 6C; scale bar = 50 μm), following the acquisition of cetuximab tolerance by CTX-DTP CRC persister cells suggested that adipocyte differentiation plays a critical role in the generation of cetuximab-tolerant clones. The upregulation of the FABP4/UCP2 axis and subsequent increase in the levels of adipogenic markers (FASN and PPAR-γ) in the CTX-DTP CRC cells upon coculture with adipocytes indicated the importance of this axis in promoting adipogenesis in persister cells during their interaction with adipocytes, leading to their transformation into treatment-tolerant persister cells in CRCs (Fig. 6D). FABP4 knockdown in CTX-DTP CRC cells co-cultured with adipocytes reduced the invasion activity of CTX-DTP CRC cells upon cetuximab treatment, suggesting that targeting FABP4 may inhibit the development of treatment tolerance (Fig. 6E; scale bar = 50 μm). Additionally, co-treatment with the FABP4 inhibitor BMS309403 and cetuximab in the co-culture system of CTX-DTP CRC cells and adipocytes decreased the invasiveness of CRC persister cells, indicating that inhibiting FABP4 disrupts adipocyte-induced cetuximab tolerance in CRC cells (Fig. 6F; scale bar = 50 μm). However, further elucidation of the detailed mechanisms and preclinical investigation of targetable FABP4/UCP2 treatment is required. Therefore, we intend to elaborate on our results regarding the mechanism and preclinical efficacy of such treatment in our subsequent in vivo study.

### Targeting FAPB4 mitigates cetuximab resistance induced by KRAS mutations in vivo

**Fig. 7:**
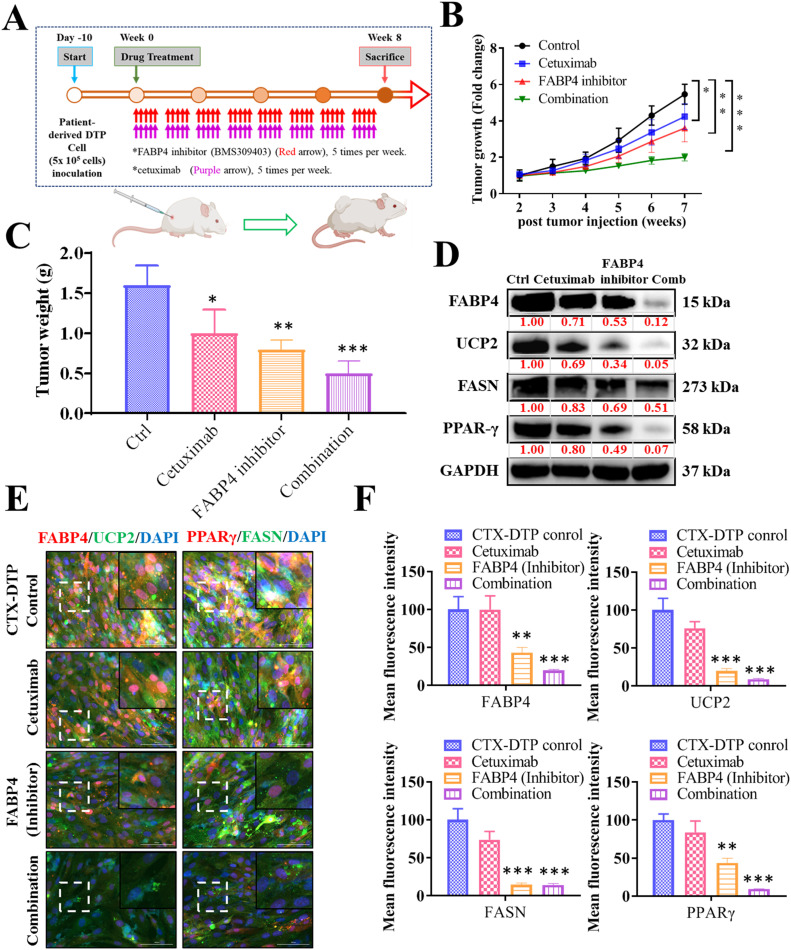
**Targeting of FABP4 mitigating cetuximab resistance induced by KRAS mutations in vivo.** The efficacy of the FABP4 inhibitor (BMS309403) in cetuximab-resistant CRC cells harbouring KRAS mutations was assessed in a xenograft mouse model established with patient-derived organoids DTP containing KRAS mutations (A). Treatment with intraperitoneal injections of cetuximab did not exhibit a significant effect on tumor growth. Conversely, treatment with BMS309403 alone or in combination with cetuximab led to a notable reduction in both tumor size and weight (B and C). Consistent with the in vitro findings, treatment with BMS309403 alone or in combination with cetuximab, but not cetuximab alone, reduced the activity of FABP4 and UCP2 as well as the levels of the FASN and PPAR-γ proteins, as indicated by the results of the immunoblotting analysis (D), immunofluorescence analysis, and subsequent quantification (E and F). *P < 0.05, **P < 0.005, and ***P < 0.001.

The effectiveness of the FABP4 inhibitor (BMS309403) in the cetuximab-resistant CRC cells harbouring KRAS mutations was evaluated in a xenograft mouse model, where tumors were generated after implantation of patient-derived organoids DTP harboring KRAS mutations (Fig. 7A). The mice that received cetuximab via intraperitoneal injection did not exhibit any change with respect to concerning tumor growth. Conversely, treatment with BMS309403 alone or in combination with BMS309403 and cetuximab led to a significant reduction in both tumor size and weight (Fig. 7B and C). Consistent with the in vitro findings, treatment with BMS309403 alone or in combination with cetuximab, but not cetuximab alone, reduced the activity of FABP4 and UCP2 and the protein levels of FASN and PPAR-γ, as indicated by the results of immunoblotting analysis (Fig. 7D), immunofluorescences analysis, and subsequent quantification (Fig. 7E and F). Together, these results indicate that BMS309403 efficiently inhibited tumor growth and overcame cetuximab insensitivity in KRAS-mutated cells by targeting the degradation of the KRAS protein.

## Discussion

CRC remains a predominant global health challenge worldwide and a leading cause of cancer-related mortality, with a considerable number of cases presenting as (mCRC), which has a poor prognosis [22]. Although treatments targeting the EGFR, such as cetuximab, have demonstrated promise, the emergence of resistance in cancer cells, which typically occurs within 8–10 months of treatment initiation [[23], [24], [25], [26]], hampers their effectiveness. This resistance presents a notable challenge with respect to managing CRC, indicating an urgent need for innovative therapeutic strategies. Numerous studies have highlighted the pivotal role of nongenetic factors, such as the TME, particularly CAAs, in fostering this resistance [26,27]. DTP cells, which are characterized by a quiescent or slow-cycling state, evolve in response to targeted therapies and persist within the TME, leading to poorer treatment outcomes [28]. The dynamic interactions within the TME, which involve adipocytes, CAFs, and TAMs, are crucial in modulating cancer cell behavior and drug sensitivity [[29], [30], [31], [32]]. Obesity is a notable risk factor for the development and progression of various cancers, including CRC. This is primarily because of the systemic effects of excess adipose tissue, which contribute to chronic inflammation, hormone imbalances, and metabolic disturbances conducive to cancer development and progression [33,34]. Within the TME, adipocytes, particularly CAAs, play a pivotal role in that they directly influence cancer cell behavior through secretion of adipokines, cytokines, and growth factors. These factors not only support tumor growth and metastasis but also foster an environment conducive to the survival and persistence of cancer cells under therapeutic stress [35,36].

The results of the current study indicate that the interaction between CAAs and CRC cells significantly contributes to the development of cetuximab resistance. Adipocytes in the TME not only foster cancer cell survival and proliferation through secretion of adipokines and inflammatory mediators but also enhance the metabolic adaptability of cancer cells by facilitating increased FA uptake and utilization [[33], [34], [35], [36]]. This metabolic reprogramming is mediated by upregulated CD36 and is critical for maintaining the viability of DTP cells under therapeutic stress, which contributes to their transient resistance against cetuximab. Upregulation of the FABP4 and UCP2 genes, which was observable in the DTP cells derived from patient-derived organoids in this study, has been identified as a key mediator of this resistance mechanism. The high expression of these genes in the adipocytes of the TME highlights their involvement in the reprogramming of lipid metabolism in CRC cells, which is a crucial factor in the development and persistence of drug resistance. This axis not only promotes the survival of DTP cells but also enables them to adversely influence the treatment landscape by modulating the cellular environment to promote cancer progression and resistance [37,38]. Furthermore, FABP4 can modulate inflammatory pathways crucial for tumor survival and proliferation under stress.

Our organoid models derived from patients with CRC who did not respond to cetuximab treatment offer a distinct and clinically relevant platform for investigating these interactions. These models accurately reflect the original tumor architecture and confirm the increased expression of EGFR and resistance-related genes (FABP4 and UCP2) in a controlled and reproducible in vitro setting. Through these models, our study demonstrated the dual role of adipocytes in the TME: providing structural support to the tumor and driving functional and phenotypic alterations in CRC cells that lead to treatment resistance. In this context, the role of FABP4 becomes crucial; FABP4 plays a pivotal role in the transportation and intracellular storage of FAs and is implicated in numerous metabolic and inflammatory pathways associated with obesity and cancer, as evident in our in vitro study. Upregulation of FABP4 in CRC cells, particularly those in proximity to adipocytes within the TME, can lead to enhanced lipid uptake and storage, which can confer metabolic advantages that foster drug tolerance and resistance. Furthermore, FABP4 can modulate inflammatory pathways crucial to tumor survival and growth under stress conditions. Given this context, targeting FABP4 presents a promising therapeutic strategy. Inhibiting the activity of FABP4 by using a specific inhibitor, such as BMS309403, could disrupt critical metabolic and inflammatory pathways, potentially reversing the adaptive resistance mechanisms observed in DTP cells, as evident in our in vivo study. By limiting the availability of essential FAs required for membrane synthesis, energy storage, and the production of signaling molecules, an FABP4 inhibitor is capable of effectively starving DTP cells of the resources necessary to maintain their drug-tolerant state. Additionally, reducing the activity of FABP4 could mitigate inflammatory signaling within the TME, further weakening the supportive environment that promotes DTP cell survival.

Our study, which employed organoid models derived from patients with CRC who did not respond to cetuximab treatment, offers unique insight into these complex interactions. These models not only replicate the intricate architecture and cellular diversity of the original tumors but also preserve the metabolic crosstalk between CRC cells and adipocytes. Thus, they serve as invaluable platforms for assessing the effectiveness of FABP4 inhibitors. Our initial findings suggest that disruption of FABP4 activity can reduce viability and enhance drug sensitivity in these organoids, indicating the potential of FABP4 inhibitors as adjuncts to current EGFR-targeted therapies for overcoming drug resistance in CRC. This enhanced understanding of the metabolic interplay among obesity, the TME, and cancer survival underscores the potential of targeting metabolic modulators such as FABP4 in the management of drug-resistant CRC. Such strategies could revolutionize treatment paradigms, potentially leading to improved outcomes for a disease largely influenced by both genetic and lifestyle factors.

## Conclusions

**Fig. 8:**
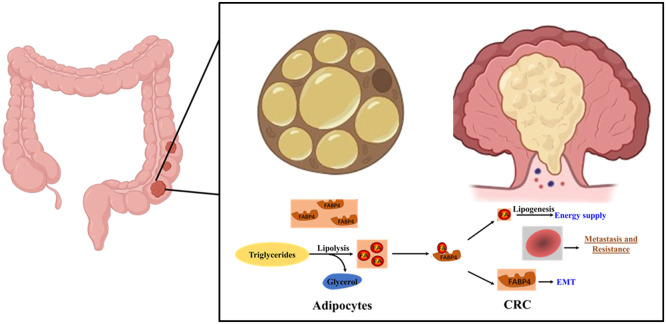
**Schematic of the role of lipid metabolism in CRC.** Elevated levels of FABP4 and UCP2 were observed in the adipocyte-rich areas, and their knockdown or the use of a pharmacological inhibitor led to changes in their expression levels, indicating targeting of CRC resistance.

As illustrated in Fig. 8, our study findings indicate the importance of the FABP4 and UCP2 axis, which constitutes a substantial oncogene pathway, and a strong correlation between their activation and suboptimal treatment outcomes. Furthermore, activation of the FABP4/UCP2 axis is prominently observed in CAAs, where it regulates complex functions in tumor progression and drug responses. These functions include the secretion of adipose FA-binding protein and the provision of metabolites and energy sources crucial to tumor proliferation. Overall, the study findings validate the concept of adipocyte–cancer cell interactions and present a therapeutic strategy for combating cetuximab-resistant cells in CRC.

## Ethics approval and consent to participate

Tissue samples were collected from adult patients undergoing surgery, in accordance with the ethical guidelines established by the Tri-Service General Hospital Institutional Review Board (TSGHIRB No.: A202405118). Our manuscript clearly indicates whether informed consent was obtained from all participating patients. Additionally, it includes a statement confirming that all participants provided written informed consent prior to their involvement in the research, as required by the ethical guidelines of the relevant Institutional Review Board or Ethics Committee overseeing the study.

## Consent for publication

Not applicable.

## Availability of data and materials

The datasets utilized and analyzed during the current study are available from the corresponding author upon reasonable request. We encourage researchers and interested parties to reach out for access to these datasets, as we are committed to promoting transparency and facilitating further research. Access to the datasets will be granted under appropriate circumstances and in accordance with any relevant data-sharing policies and ethical considerations.

## Abbreviation lists

Cancer-associated adipocytes (CAAs); Cancer-associated fibroblasts (CAFs); colorectal cancer (CRC); cluster of differentiation 36 (CD36); drug-tolerant persisters (DTPs); epidermal growth factor receptor (EGFR); fatty acid-binding protein 4 (FABP4); tumor microenvironment (TME); tumor-associated macrophages (TAMs).

## Funding

This current study was backed by the National Science Council of Taiwan: Jo-Ting Tsai (MOST111-2314-B038-073-MY3) and Chi-Tai Yeh (MOST111-2314-B038-139; NSTC113-2314-B038-125). This research was backed by a grant from the National Taiwan University Hospital-Taipei Medical University Joint Research Program (NTUH-TMU Joint Research Program) offered to Chi-Tai Yeh (111-TMU303).

## CRediT authorship contribution statement

**Yi-Chiao Cheng:** Data curation, Conceptualization. **Ming-Yao Chen:** Investigation, Formal analysis. **Vijesh Kumar Yadav:** Investigation, Funding acquisition, Conceptualization. **Narpati Wesa Pikatan:** Methodology, Formal analysis. **Iat-Hang Fong:** Formal analysis, Data curation. **Kuang-Tai Kuo:** Conceptualization, Data curation, Formal analysis, Funding acquisition, Investigation. **Chi-Tai Yeh:** Visualization, Validation, Supervision. **Jo-Ting Tsai:** Visualization, Validation, Supervision, Resources, Project administration.

## Declaration of competing interest

The authors declare no conflict of interest regarding the publication of this paper.

## Appendix Supplementary materials

Image, application 1
