# Supplementary material for: Targeting FABP4/UCP2 axis to overcome cetuximab resistance in obesity-driven CRC with drug-tolerant persister cells
Source: Transl Oncol. 2025 Jan 16;53:102274. doi: 10.1016/j.tranon.2025.102274 (PMC11787020; doi:10.1016/j.tranon.2025.102274)
Supplement: Supplementary file 1 [file mmc1.docx]

**SUPPLEMENTARY DATA**

**Targeting FABP4/UCP2 Axis to Overcome Cetuximab Resistance in Obesity-Driven CRC with Drug-Tolerant Persister Cells**

Yi-Chiao Cheng^1,2^, Ming-Yao Chen^3,4^, Vijesh Kumar Yadav^3,4^, Narpati Wesa Pikatan^5^, Iat-Hang Fong^6,7^, Kuang-Tai Kuo^8^, Chi-Tai Yeh^6,7^*, and Jo-Ting Tsai^1,9,10^*

1. Graduate Institute of Clinical Medicine, College of Medicine, Taipei Medical University, Taipei, Taiwan 110.
2. Division of Colon and Rectal Surgery, Department of Surgery, Tri-Service General Hospital, National Defense Medical Center, Taipei, Taiwan.
3. Division of Gastroenterology and Hepatology, Department of Internal Medicine, School of Medicine, College of Medicine, Taipei Medical University, Taipei, Taiwan.
4. Division of Gastroenterology and Hepatology, Department of Internal Medicine, Shuang Ho Hospital, New Taipei City, Taiwan.
5. Division of Urology, Department of Surgery, Faculty of Medicine, Universitas Gadjah Mada, Yogyakarta, Indonesia 55281.
6. Department of Medical Research & Education, Taipei Medical University–Shuang Ho Hospital, New Taipei City, Taiwan 235.
7. Continuing Education Program of Food Biotechnology Applications, College of Science and Engineering, National Taitung University, Taitung 95092, Taiwan.
8. Department of Surgery, Division of Thoracic Surgery, Taipei Medical University Shuang-Ho Hospital, New Taipei City 23561, Taiwan
9. Department of Radiology, School of Medicine, College of Medicine, Taipei Medical University, Taipei, Taiwan.
10. Department of Radiology, Taipei Medical University–Shuang Ho Hospital, New Taipei City, Taiwan.

*Authors to whom correspondence should be addressed.

*Prof. Chi-Tai Yeh, PhD. Department of Medical Research & Education, Taipei Medical University - Shuang Ho Hospital, New Taipei City, 235, Taiwan. Phone: 886-2-2490088 ext. 8885; FAX: 886-2-2248-0900. E-mail: [ctyeh@s.tmu.edu.tw](mailto:ctyeh@s.tmu.edu.tw)

*Prof. Jo-Ting Tsai, MD, PhD, Department of Radiation Oncology, Cancer Center, Taipei Medical University–Shuang Ho Hospital, New Taipei City Taiwan 23561; Phone: 886-2-2490088 ext. 8881; FAX: 886-2-2248-0900. E-mail: [10576@s.tmu.edu.tw](mailto:10576@s.tmu.edu.tw)

**Supplementary Table S1.** List of antibodies and dilution used.


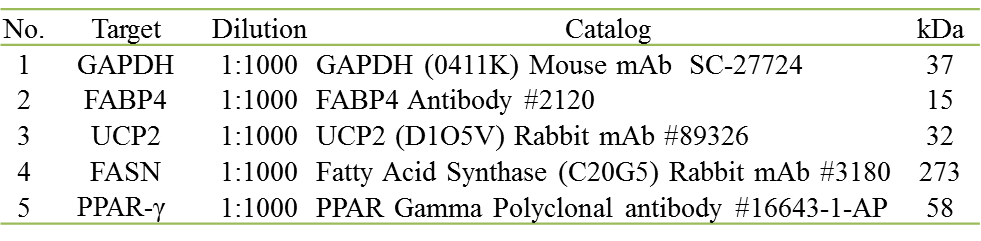


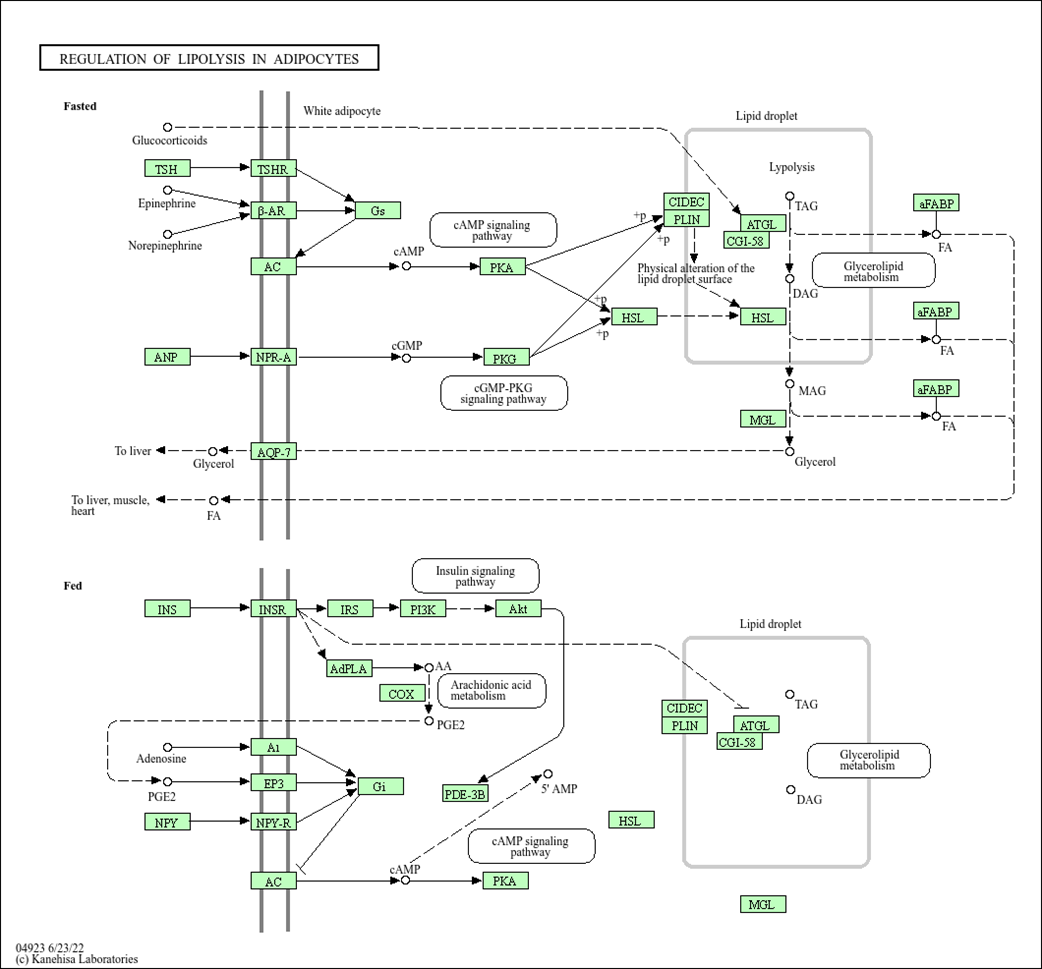


**Supplementary Figure S1:** KEGG pathway analysis of differentially expressed genes (DEGs) between cetuximab non-responders and responders’ highlights pathways associated with the regulation of lipolysis in adipocytes, underscoring the metabolic alterations in the tumor microenvironment that contribute to therapy resistance


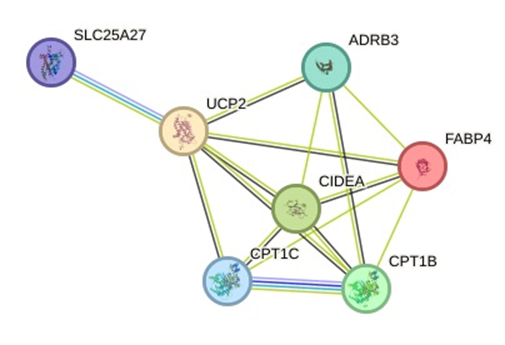


**Supplementary Figure S2:** STRING protein-protein interaction analysis showing a strong association between FABP4 and UCP2, reinforcing their functional interplay in metabolic reprogramming and drug resistance mechanisms in cetuximab-resistant colorectal cancer.


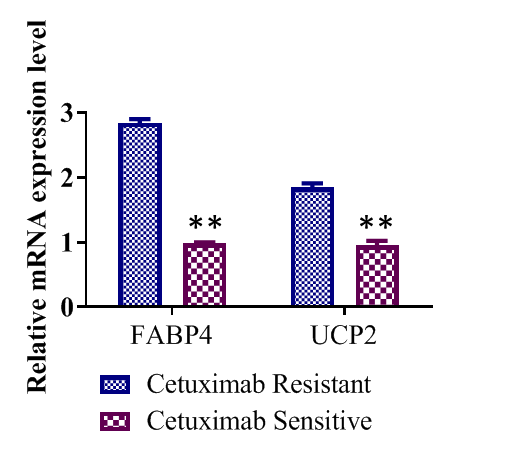


**Supplementary Figure S3:** Bar plot illustrating qRT-PCR analysis results for FABP4 and UCP2 expression in cetuximab-resistant versus sensitive samples, validating their differential expression and highlighting their potential roles as key mediators of resistance.


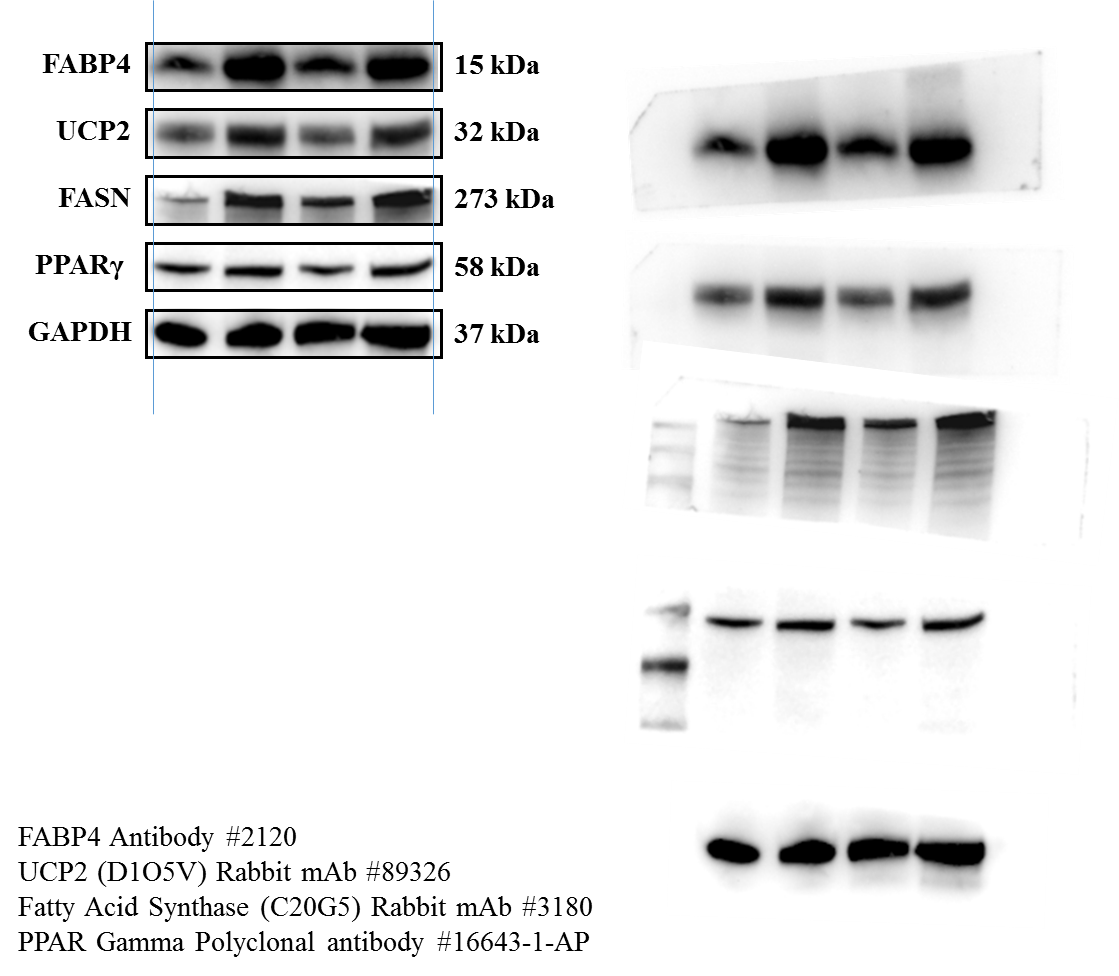


**Supplementary Western Blot.** Full-size blots of Figure 5B


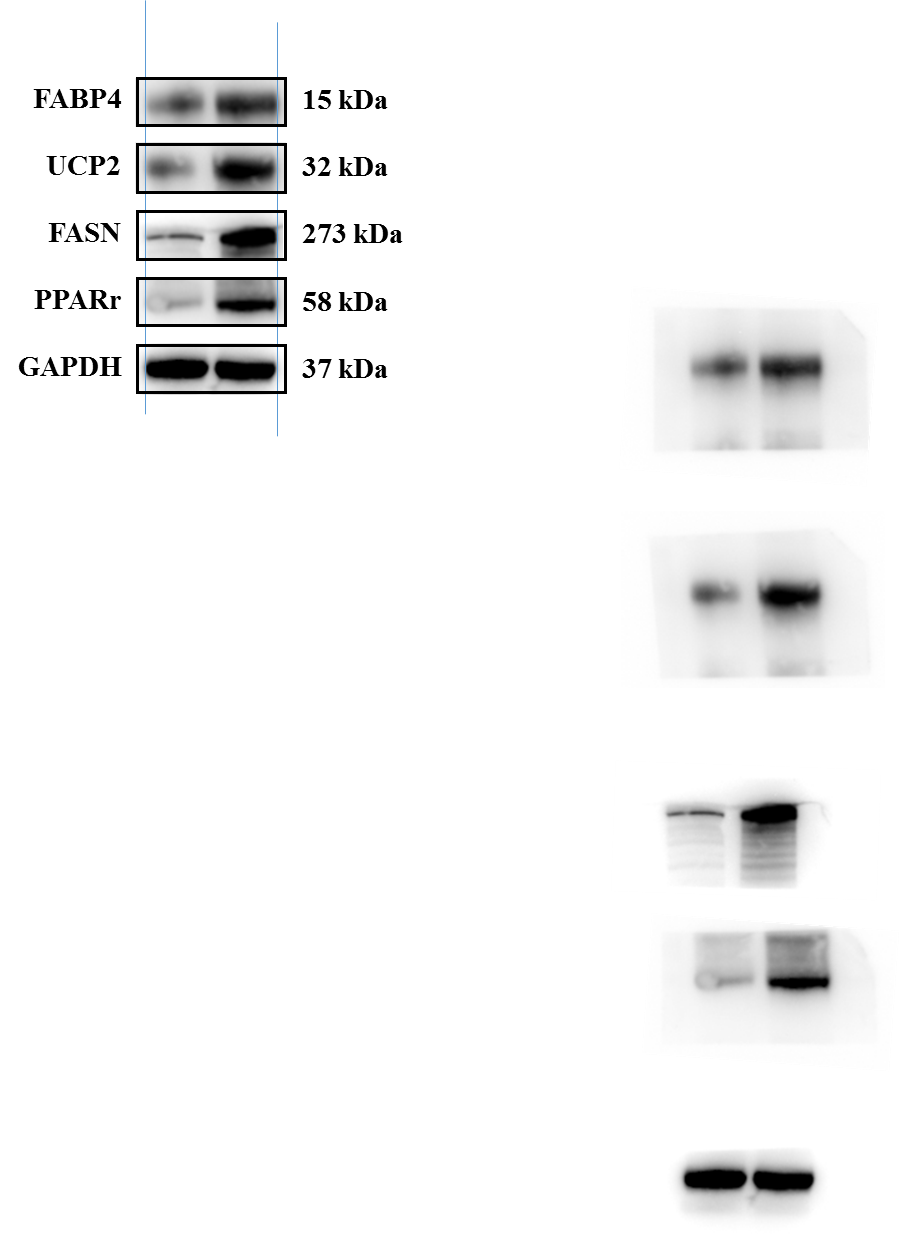


**Supplementary Western Blot.** Full-size blots of Figure 6D


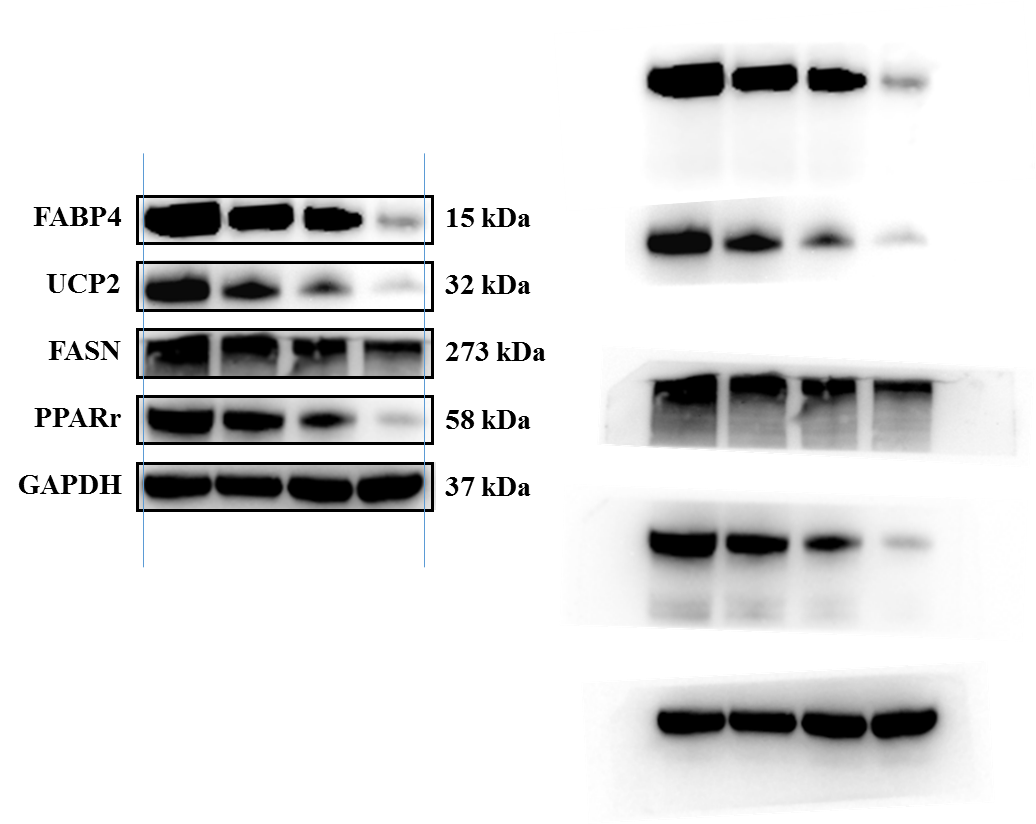


**Supplementary Western Blot.** Full-size blots of Figure 7D
